# Protection of the Transplant Kidney from Preservation Injury by Inhibition of Matrix Metalloproteinases

**DOI:** 10.1371/journal.pone.0157508

**Published:** 2016-06-21

**Authors:** Michael A. J. Moser, Steve Arcand, Han-Bin Lin, Chris Wojnarowicz, Jolanta Sawicka, Tamalina Banerjee, Yigang Luo, Gavin R. Beck, Patrick P. Luke, Grzegorz Sawicki

**Affiliations:** 1 Department of Surgery and Saskatchewan Renal Transplant Program, Saskatoon, Saskatchewan; 2 Department of Pharmacology, University of Saskatchewan, Saskatoon, Saskatchewan, Canada; 3 University of Ottawa Heart Institute, Ottawa, Ontario, Canada; 4 Prairie Diagnostic Services, Department of Veterinary Pathology, University of Saskatchewan, Saskatoon, Saskatchewan, Canada; 5 Department of Laboratory Medicine and Pathology, Saskatoon Health Region, Saskatoon, Saskatchewan, Canada; 6 Multi Organ Transplant Program, London Health Sciences Centre, London, Ontario, Canada; 7 Department of Clinical Chemistry, Medical University of Wroclaw, Wroclaw, Poland; Florida State University, UNITED STATES

## Abstract

**Background:**

Matrix metalloproteinases (MMPs), particularly MMP-2 and MMP-9, play an important role in ischemic injury to the heart, yet it is not known if these MMPs are involved in the injury that occurs to the transplant kidney. We therefore studied the pharmacologic protection of transplant kidneys during machine cold perfusion.

**Methods:**

Human kidney perfusates were analyzed for the presence of injury markers such as cytochrome c oxidase, lactate dehydrogenase, and neutrophil-gelatinase associated lipocalin (NGAL), and MMP-2 and MMP-9 were measured. The effects of MMP inhibitors MMP-2 siRNA and doxycycline were studied in an animal model of donation after circulatory determination of death (DCDD).

**Results:**

Markers of injury were present in all analyzed perfusates, with higher levels seen in perfusates from human kidneys donated after controlled DCDD compared to brain death and in perfusate from kidneys with delayed graft function. When rat kidneys were perfused at 4°C for 22 hours with the addition of MMP inhibitors, this resulted in markedly reduced levels of MMP-2, MMP-9 and analyzed injury markers.

**Conclusions:**

Based on our study, MMPs are involved in preservation injury and the supplementation of preservation solution with MMP inhibitors is a potential novel strategy in protecting the transplant kidney from preservation injury.

## Introduction

In an effort to increase the number of kidneys available for transplantation in the face of ongoing donor organ shortage, the use of kidneys from more marginal donors has been increasing. This includes the use of kidneys from older donors and those with hypertension [Expanded Criteria Donors (ECD)] as well as Donation after Circulatory Determination of Death donors (DCDD). Unfortunately, both ECD and DCDD donated kidneys are associated with a higher rate of Delayed Graft Function (DGF) and poorer function at one year [1]. Although DCDD donation has increased the numbers of donor kidneys as much as 30% in some programs, this comes with a disadvantage- delayed graft function, poorer long-term function, and increased risk of rejection compared to donation after brain death donation [2,3]. Efforts to minimize the warm ischemic damage and preserve glomeruli would be very worthwhile, especially when one considers that with time, there is steady drop off of glomeruli and kidney transplants have a median graft survival of about 10 years.

Machine cold perfusion of transplant kidneys has shown benefit in terms of both early function and long term function [4,5,6] and this benefit may be greater in more marginal kidneys and those obtained from donation after cardiac death donors [7]. Our group’s previous studies on the heart have shown that MMP-2 is released [8] and also contributes to the injury that occurs to the ischemic heart [9]. Furthermore, the use of MMP inhibitors in hearts subjected to ischemic injury protects the heart from damage [10,11].

MMP-2 and MMP-9 have been shown to be involved in acute and chronic renal injury along the spectrum of basement membrane damage, to tubular atrophy, to fibrosis, to outright renal failure [12,13]. MMPs have also been shown to play an important role in injury to the transplanted kidney. MMP’s are increased in patients with chronic antibody mediated rejection and because of the role of MMPs in the fibrotic renal diseases, MMPs have been suggested as a possible common pathway for chronic allograft nephropathy in the transplanted kidney [14].

Finally, MMP-2 has been shown to be involved in renal ischemia-reperfusion injury in an animal model whereby warm ischemia was induced in situ for 30 to 120 minutes in an MMP-2 deficient transgenic mouse model [15]. The degree of acute tubular injury, necrosis, apoptosis and renal dysfunction was markedly less in the MMP-2 deficient transgenic mice compared to that seen in the wild type mice. Similar mechanisms of injury may be at work in the ischemic cold-perfused kidney, and if this is the case, MMPs should be a clinically useful target for pharmacologic protection of the transplant kidney from preservation injury.

In this study, we examined the perfusate from human perfused kidneys and documented the presence of MMPs. We then developed an experimental animal model of machine cold perfusion to study the effect of inhibition of MMPs.

## Results

### Release of injury markers from human kidneys during preservation

**Fig 1:**
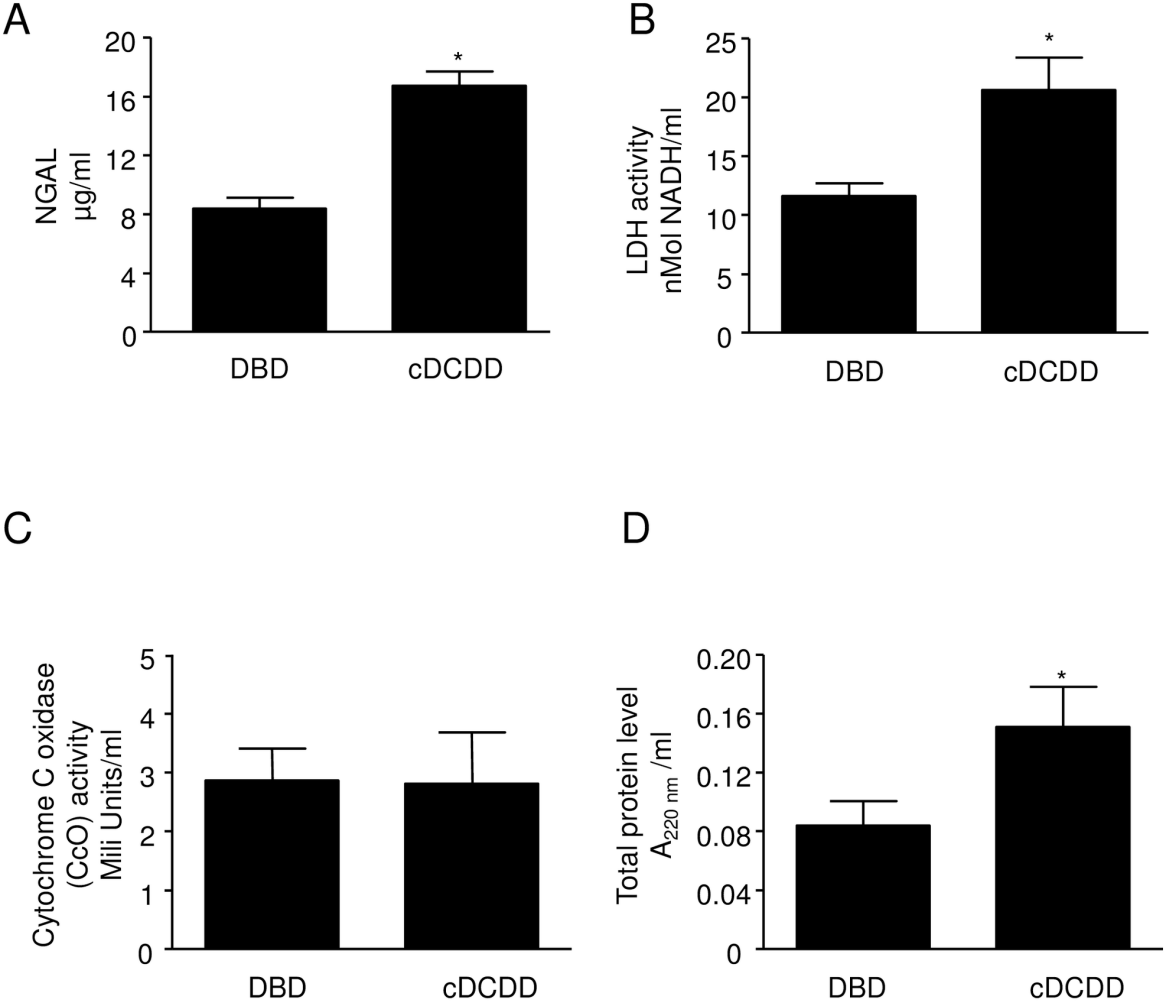
Comparison of injury markers and total protein in perfusate from DBD (n = 17) versus cDCDD (n = 9) human donors. A: NGAL activity B: LDH activity C: Cytochrome c oxidase activity D: Total protein level Error bars represent Standard Error of the Mean (SEM). * p<0.05 using Student’s T test. DBD = Donation after Brain Death cDCDD = controlled Donation after Circulatory Determination of Death LDH = lactate dehydrogenase NGAL = neutrophil gelatinase associated lipocalin

Twenty-four perfusates of human transplant kidneys obtained from two transplant programs between July 1, 2012 and June 30, 2013 were studied (Table 1). NGAL, LDH and CcO (markers of kidney injury) [16,17,18] activity was detected in all collected samples. The levels of total protein, NGAL and LDH in perfusates from cDCDD kidneys were significantly higher than that found in perfusates from DBD kidneys (Fig 1A, 1B and 1D). In contrast the activity of CcO (Fig 1C), a marker of mitochondrial injury, was similar for the two groups in spite of the shorter preservation time for cDCDD kidneys (11.6 h vs. 17.3 h). Since CcO in the perfusate increases with time in our animal studies, we suspect that CcO in perfusate from cDCDD kidneys would be markedly higher than that for DBD if cDCDD kidneys were preserved for equivalent amounts of time.

**Table 1:** Demographic information for 24 perfusates obtained from perfusion of human kidneys destined for transplantation.

	Donation after Brain Death (DBD)(n = 15)	Donation after Circulatory Death (cDCDD)(n = 9)	p-value
Donor age (yr (95% CI))	50.8 (43.1,58.5)	40.0 (32.1,47.9)	0.08
Donor gender (M:F)	4:11	7:2	0.04*
Kidney side (R:L)	10:5	5:4	0.91
Hours on machine cold perfusion (95%CI)	15.9 (13.1,18.8)	10.3 (7.9,12.9)	0.02*
Cold Ischemic Time (CIT)	17.3 (14.2,20.4)	11.6 (9.0,14.2)	0.02*
Delayed Graft Function (DGF)	1/15 (7%)	4/9 (45%)	**0.03**#
Slow Graft Function (SGF)	1/15 (7%)	8/9 (89%)	**0.01**
Primary Non Function (PNF)	0	0	

CI = 95% Confidence Interval.

* calculated using two sided t-test.

^#^ calculated using chi-squared test.

### MMPs in perfusates

**Fig 2:**
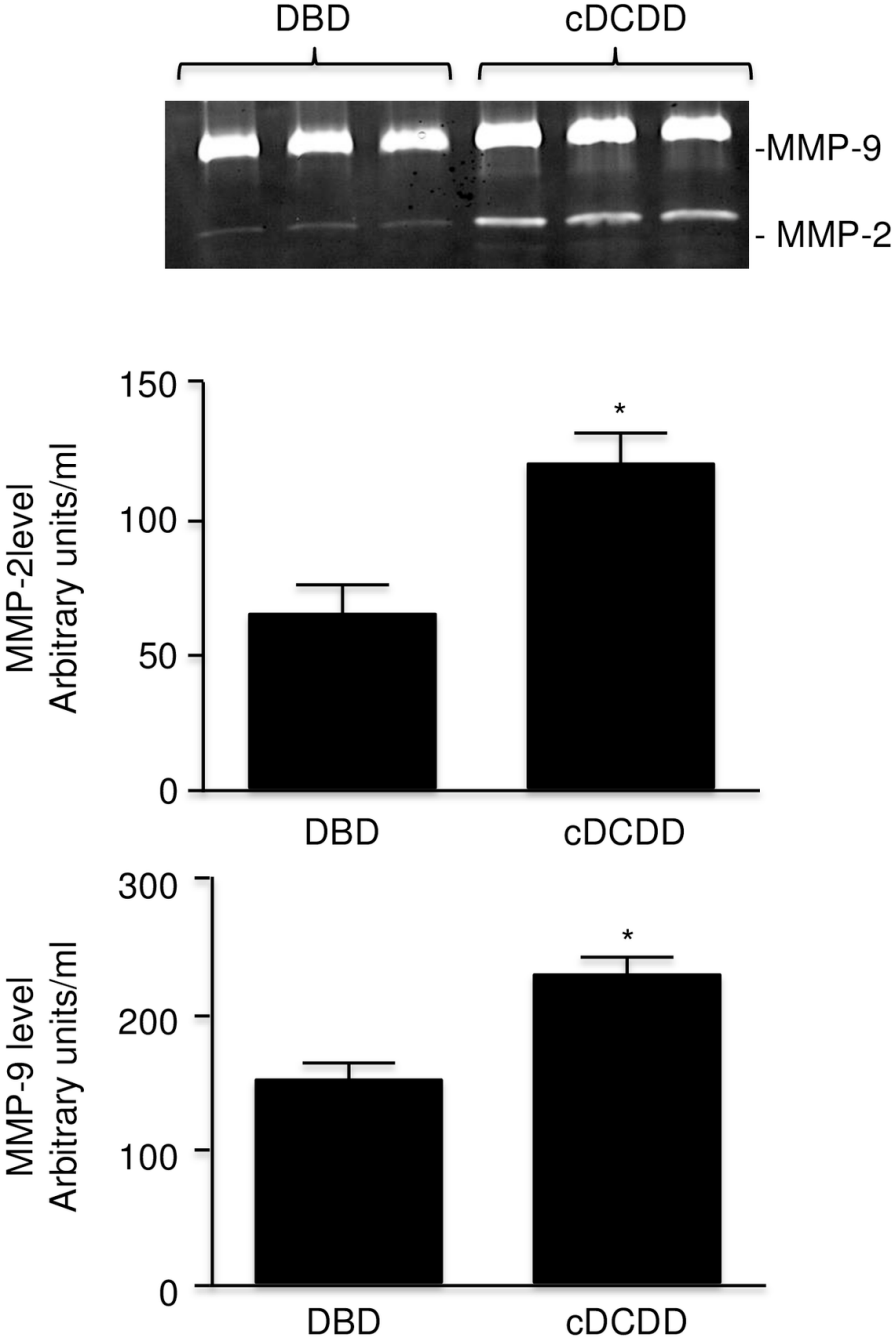
Comparison of MMP-9 and MMP-2 levels in perfusates from kidneys from DBD (n = 15) and cDCDD (n = 9) human donors. Error bars represent Standard Error of the Mean (SEM). * p<0.05 using Student’s T test. DBD = Donation after Brain Death cDCDD = controlled Donation after Circulatory Determination of Death MMP = matrix metalloproteinase.

Analysis for gelatinolytic activity (zymography) showed that all perfusates containedMMP-2 and MMP-9 (molecular weights of 72 and 92 kDa respectively) (Fig 2). The levels of both MMPs were significantly higher in perfusates from cDCDD donors than from DBD donors (Fig 2).

### DGF and MMP levels

**Fig 3:**
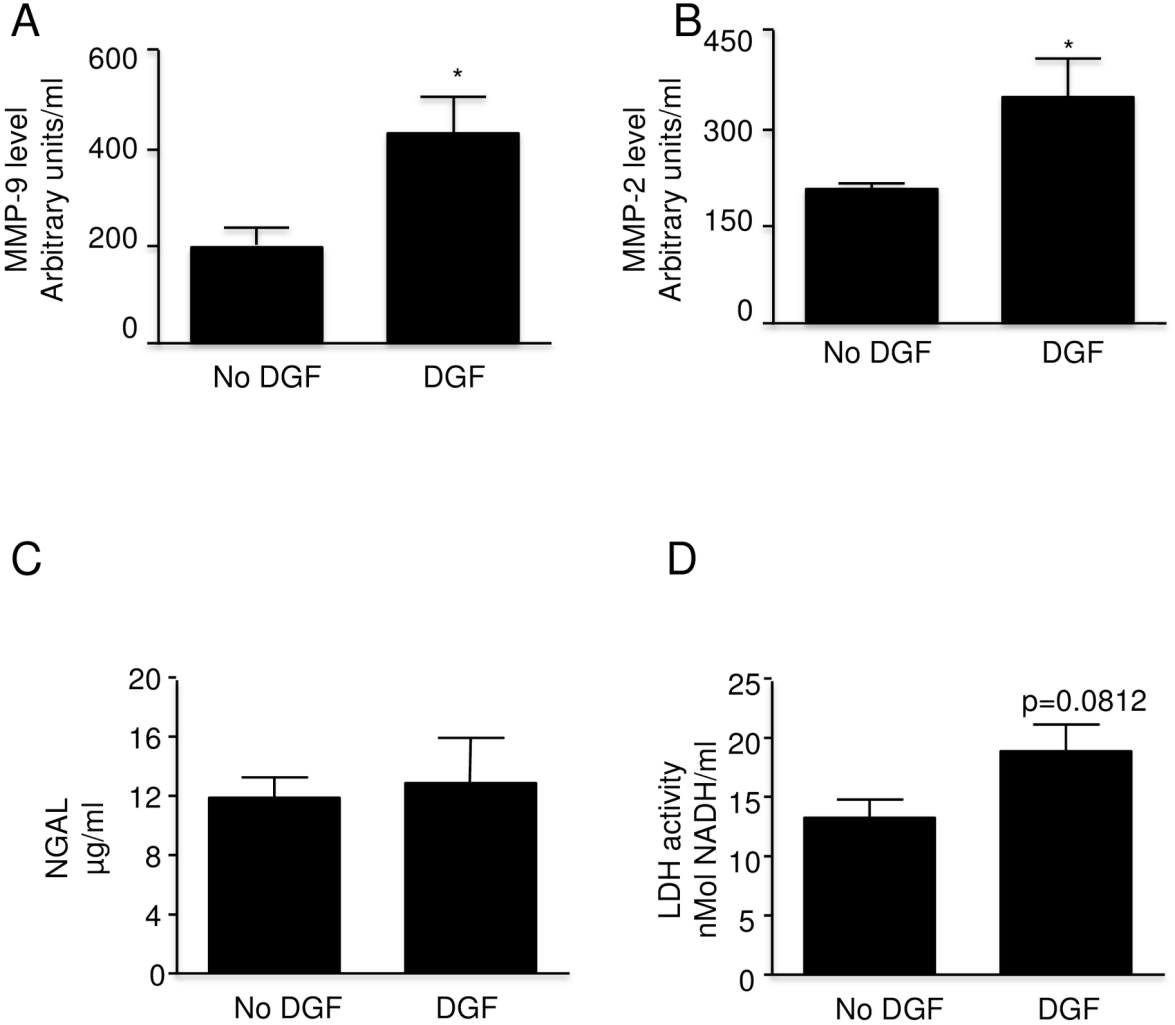
Matrix Metalloproteinase levels and injury markers in perfusate from clinically transplanted kidneys with DGF (n = 5) and without DGF (n = 19). A: MMP-9 level B: MMP-2 level C: NGAL levelD: LDH activity Error bars represent Standard Error of the Mean (SEM). * p<0.05 using Student’s T test. DGF = Delayed Graft Function MMP = matrix metalloproteinase NGAL = neutrophil gelatinase associated lipocalin.

Delayed graft function (DGF) was associated with significantly increased MMP-9 and MMP-2 levels (Fig 3A and 3B). In contrast to MMP activities the levels of NGAL and LDH (markers of kidney injury) were not significantly different in both analyzed groups (Fig 3C and 3D).

### Microscopic evaluation of animal model of cold perfusion

**Fig 4:**
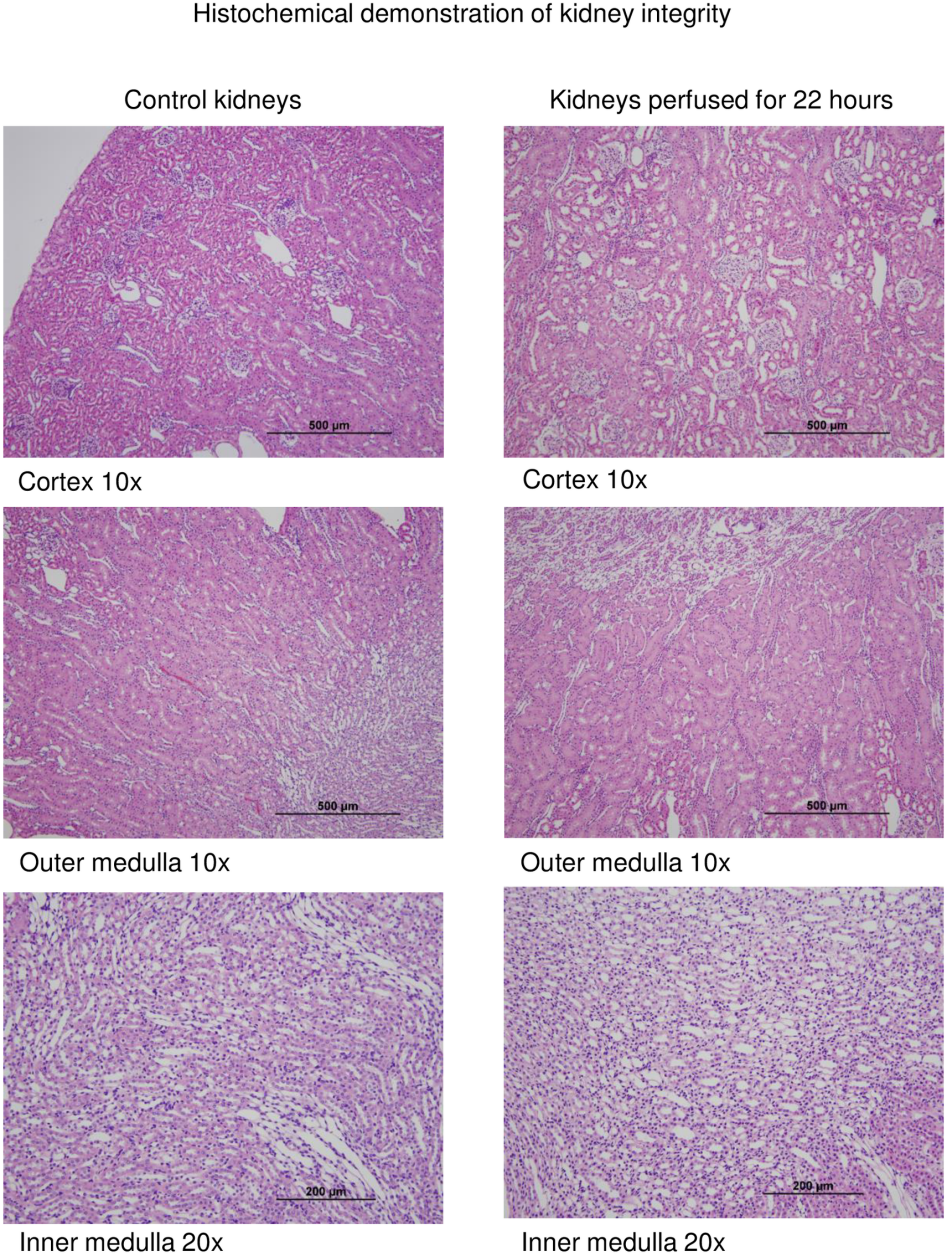
Light microscopy (H&E stain) of representative rat kidney at initial flush and after 22 hours of machine cold perfusion (n = 5 per time point).

Rat kidneys (n = 5 per time point) demonstrated preservation of gross architecture under light microscopy in kidneys cold perfused for 22 hours versus those who were flushed and fixed immediately (Fig 4).

### NGAL, LDH and CcO in perfusate from machine cold perfused rat kidneys

**Fig 5:**
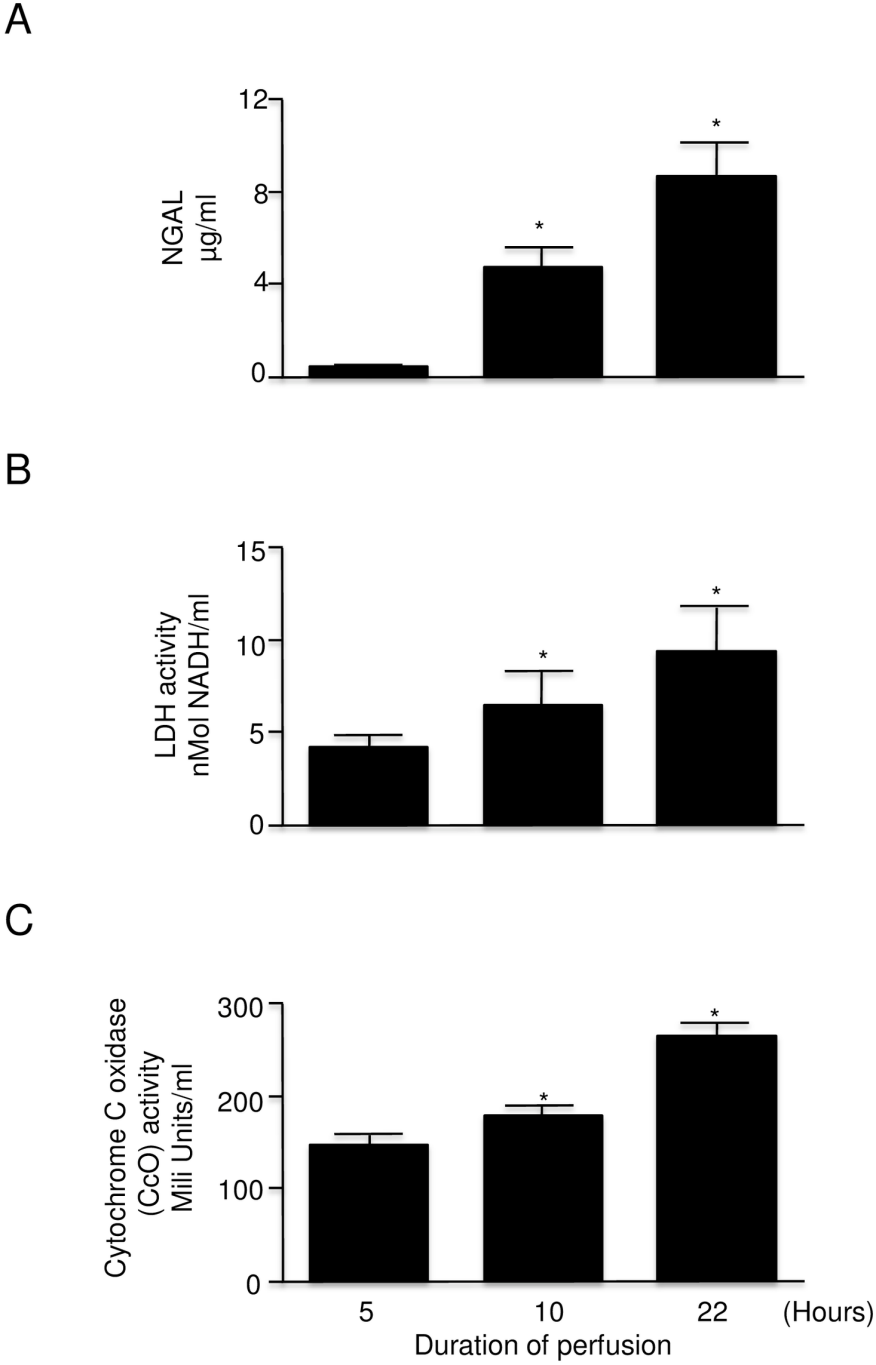
Injury markers in the perfusate of rat kidney with warm ischemic injury undergoing machine cold perfusion at 5, 10, and 22 hours (n = 4–7 each). A: NGAL levelB:LDH activityC: Cytochrome c oxidase activity Error bars represent Standard Error of the Mean (SEM). * p<0.05 using Student’s T test. LDH = lactate dehydrogenase NGAL = neutrophil gelatinase associated lipocalin.

Similar to what was observed in human kidney perfusion, NGAL, LDH and CcO were observed in the perfusate from machine cold perfusion preserved rat kidneys (Fig 5A and 5B). The release of analyzed markers was positively correlated with the increasing time of cold perfusion (Fig 5A, 5B and 5C). After 22 h of kidney perfusion the level of NGAL was approximately 10 fold higher than after 5 h of perfusion (Fig 5A). LDH activity and CcO levels were increased also but to a lesser degree (Fig 5B and 5C).

### Release of MMPs during cold perfusion

**Fig 6:**
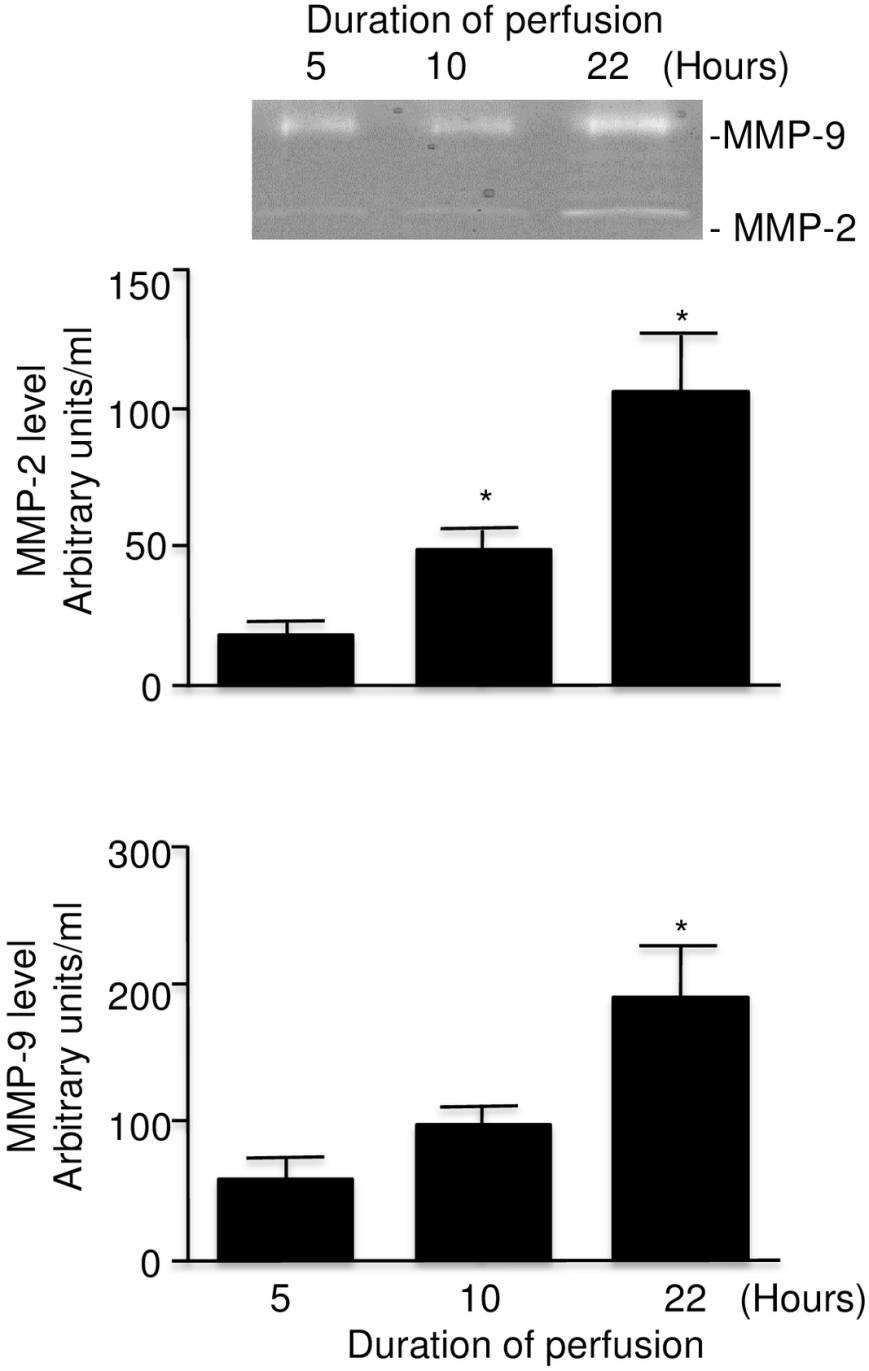
Comparison of MMP-9 and MMP-2 levels at 5, 10, and 22 hours in perfusates from rat kidney with warm ischemic injury undergoing machine cold perfusion (n = 7). Insert: representative zymography with MMP-9 and MMP-2 activitiesError bars represent Standard Error of the Mean (SEM). * p<0.05 compared to 5 hours using Student’s T test.

Analysis of perfusates for MMP-9 and MMP-2 activities (molecular weight 92 and 72 kDa respectively) showed higher activities after 22 hours of perfusion in comparison to 5 hours of perfusion. Levels of all analyzed parameters were positively correlated with the duration of cold perfusion (Fig 6).

### Evaluation of rat kidney by electron microscopy (EM)

**Fig 7:**
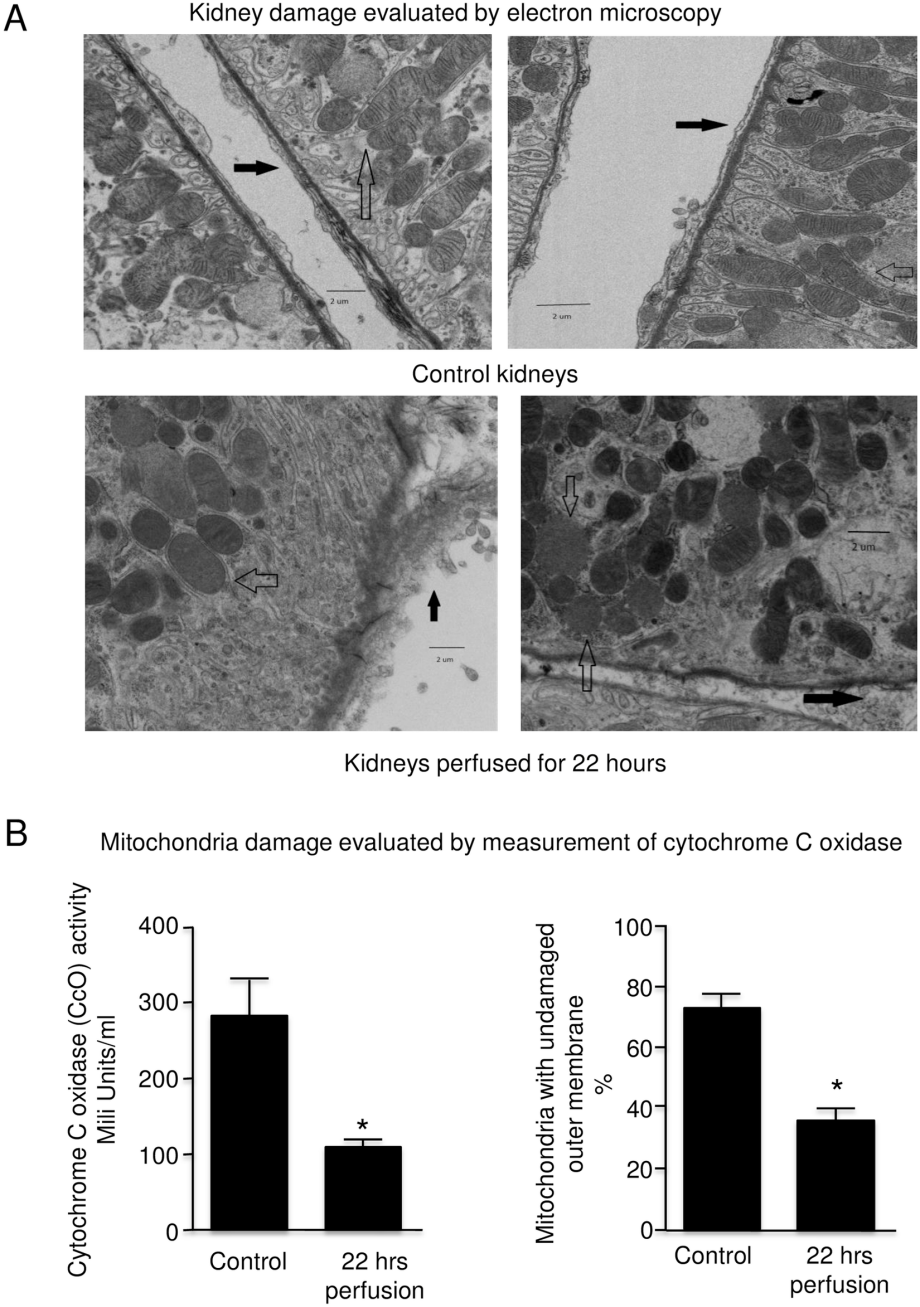
Representative electron micrographs of rat kidneys and comparison of mitochondrial damage at the time of retrieval and after 22 hours of machine cold perfusion. (5K magnification). A: Clear arrows = mitochondrial swelling and loss of detail at 22 hours Dark arrows = swelling and detachment of the tubular endothelium at 22 hours. **B (n = 4).** Left panel = cytochrome c oxidaseRight panel = percent undamaged outer membrane of mitochondria. Error bars represent Standard Error of the Mean (SEM). * p<0.05 using Student’s T test.

Changes were seen on EM as shown in Fig 7A. After 22 hours, mitochondrial swelling and loss of detail in the inner cristae was seen in all sections. There was swelling and detachment of tubule basolateral membrane connective tissues.

Analysis of activities CcO and mitochondrial outer membrane integrity in kidney *tissue* after 22 hours of cold perfusion revealed a marked decrease in activity of approximately 60% and 50% in mitochondrial integrity (Fig 7B). These results correlate to the EM appearance where roughly half of the mitochondria were visibly injured (Fig 7A). Note that the *tissue* levels of CcO are inversely proportional to the *perfusate* levels of CcO seen in Fig 5.

### Selective inhibition of MMP-2 synthesis

**Fig 8:**
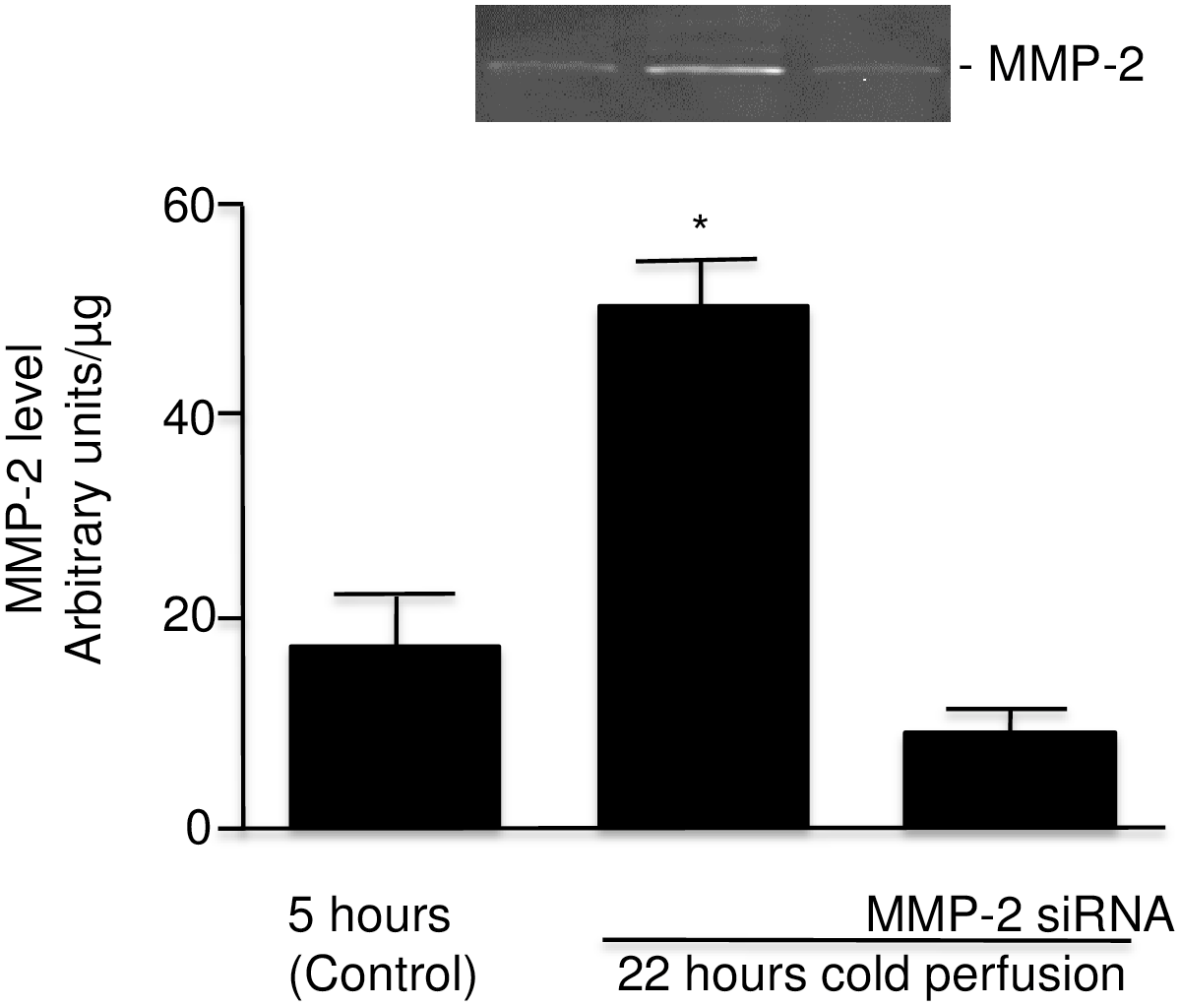
MMP-2 expression in perfusate from rat kidney cold perfused with KPS-1 solution with MMP-2 siRNA added (n = 4). Insert: a representative zymography for MMP-2. Error bars represent Standard Error of the Mean (SEM). * p<0.05 using Student’s T test. MMP = matrix metalloproteinase.

**Fig 9:**
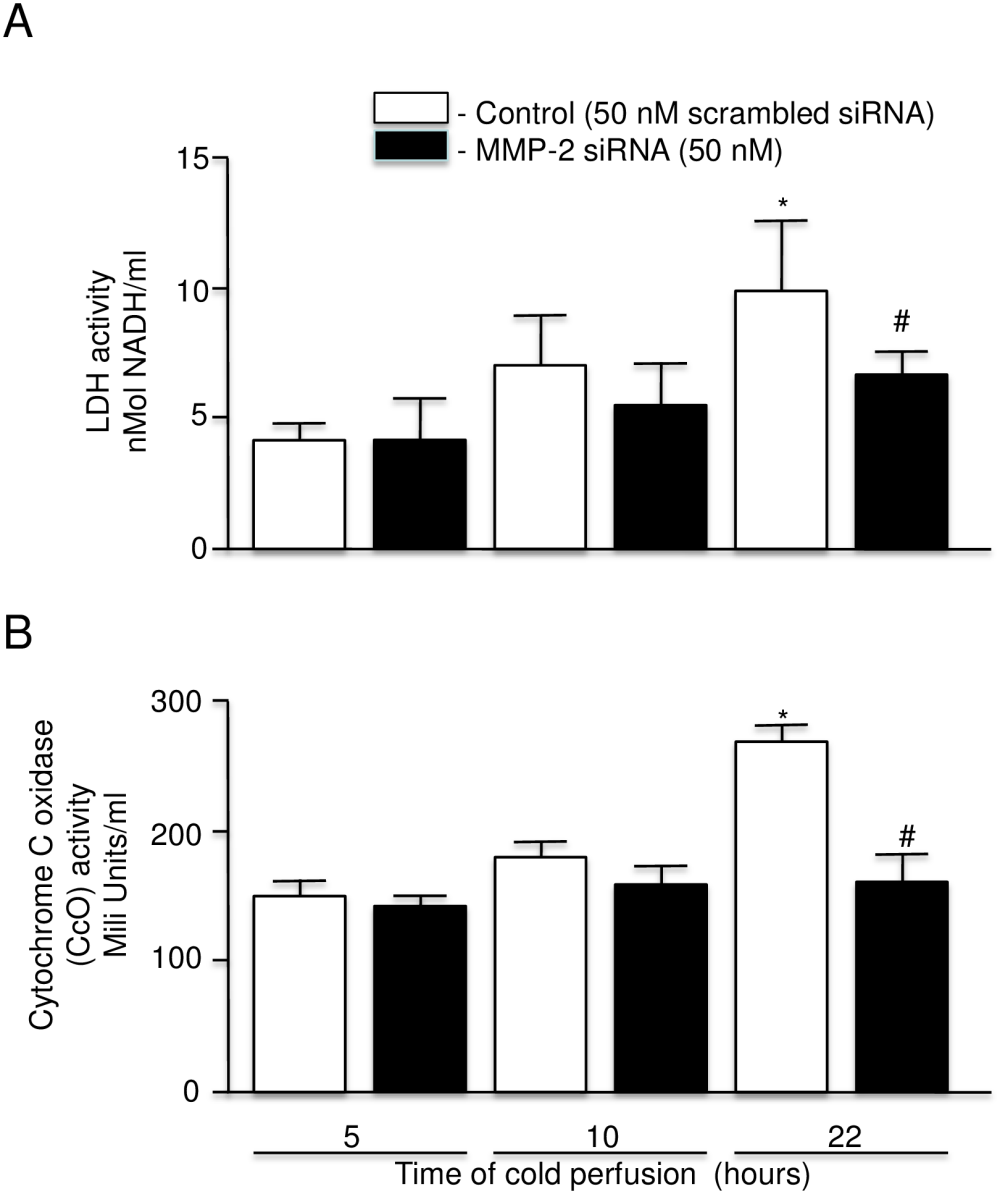
LDH activity and Cytochrome c oxidase level in perfusate of rat kidney with warm ischemic injury at 5, 10, and 22 hours perfused with KPS-1 with scrambled siRNA versus KPS-1 with siRNA 50 nM (n = 4–7 each). A: LDH activity. B: Cytochrome c oxidase level. Error bars represent Standard Error of the Mean (SEM). * p<0.05 compared to 5 hours using Student’s T test. # p<0.05 compared to 22 hours using Student’s T test.

Due to the lack of specific and selective inhibitors to MMP-9, only selective inhibition of MMP-2 (by MMP-2 siRNA) could be investigated. The level of MMP-2 (72 kDa form) in perfusate after 22 hours of cold perfusion was approximately 3 fold higher than at the time of the first perfusate sample at 5 hours (Fig 8). In contrast the level of MMP-2 in perfusates from kidneys perfused for 22 hours in the presence of MMP-2 siRNA was similar to the control level (Fig 8). Also noted were lower levels of LDH and CcO in the perfusate at 22 hours with levels similar to those for kidneys perfused for 5 hours (Fig 9A and 9B).

### Effect of DOXY on protein release from rat kidney during cold perfusion

In addition to its antibacterial activity, DOXY has also been shown to have inhibitory effect against cardiac MMP-9 and MMP-2 [5,8,11,19].

**Fig 10:**
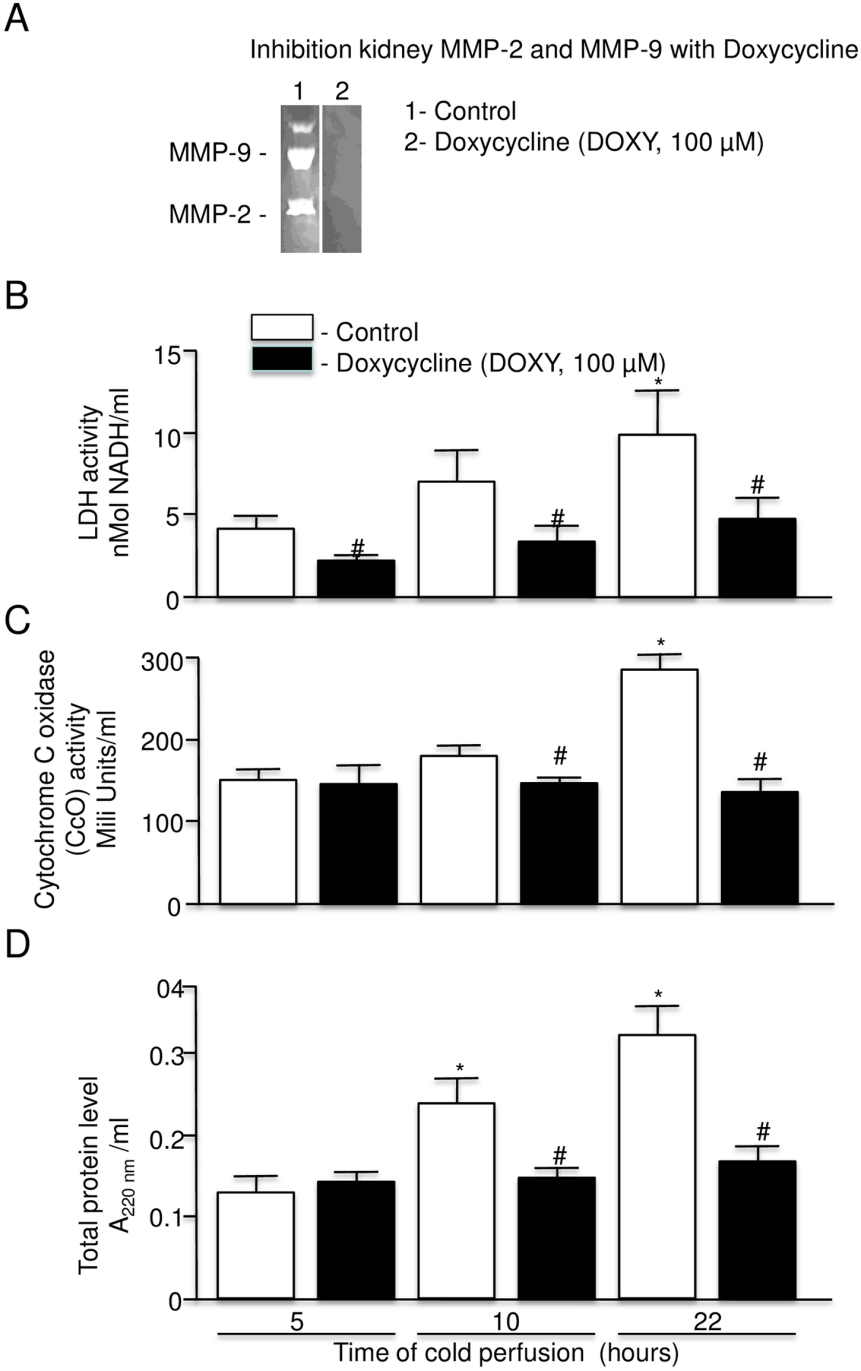
Effect of doxycycline on LDH, cytochrome c oxidase, and total protein release. A: Confirmation of inhibition of MMP-2 and MMP-9 activity by doxycycline using zymography. B: LDH activity in perfusate of rat kidney with warm ischemic injury at 5, 10, and 22 hours perfused with KPS-1 with and without doxycycline (n = 7 each). C: Cytochrome c oxidase level in perfusate of rat kidney with warm ischemic injury at 5, 10, and 22 hours perfused with KPS-1 with and without doxycycline (n = 7 each). D: Total protein release in perfusate of rat kidney with warm ischemic injury at 5, 10, and 22 hours perfused with KPS-1 with and without doxycycline (n = 6 each). Error bars represent Standard Error of the Mean (SEM). * p<0.05 compared to 5 hours using Student’s T test.# p<0.05 compared to 22 hours using Student’s T test.KPS-1 = Kidney Preservation Solution 1MMP = matrix metalloproteinase.

Analysis of the inhibitory potential of DOXY on kidney MMP-9 (92 kDa) and MMP-2 (72 kDa) showed complete inhibition of the gelatinolytic activity of both enzymes in gel 2 (DOXY) (Fig 10A).

Perfusion of rat kidney with KPS-1 solution containing DOXY showed decreased levels of LDH and CcO in the perfusate and the level of both enzymes after 22 hours of perfusion was similar to the level after 5 hours of perfusion (Fig 10B and 10C). In the case of LDH, a significant protective effect was already observed after 5 hours of perfusion (Fig 10B).

Protein release into the perfusate by the kidney was also inhibited by DOXY and was similar to the control level throughout the duration of the perfusion (Fig 10D).

### Effect of MMP inhibition on NGAL level

**Fig 11:**
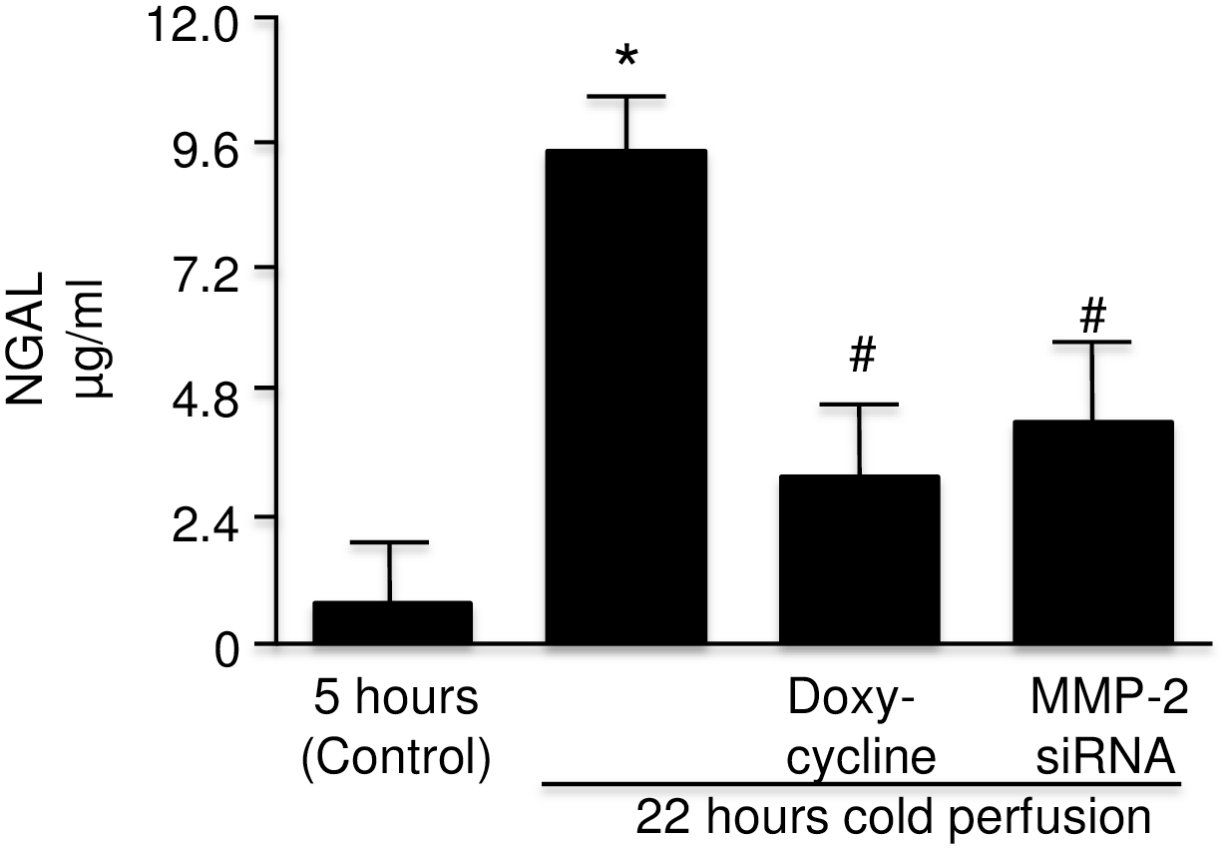
NGAL in perfusate of rat kidney with warm ischemic injury at 22 hours with KPS-1 and KPS-1 supplemented with doxycycline and MMP-2 siRNA compared to control (5 hours) (n = 4). Error bars represent Standard Error of the Mean (SEM). * p<0.05 compared to 5 hours using Student’s T test.# p<0.05 compared to 22 hours using Student’s T test.KPS-1 = Kidney Preservation Solution 1MMP = matrix metalloproteinaseNGAL = neutrophil gelatinase associated lipocalin.

The levels of NGAL observed after 22 hours of cold perfusion with DOXY or MMP-2 siRNA were markedly less than that found in perfusate without additives at 22 hours (Fig 11).

### Effect of MMP inhibition on rat kidney mitochondrial integrity

**Fig 12:**
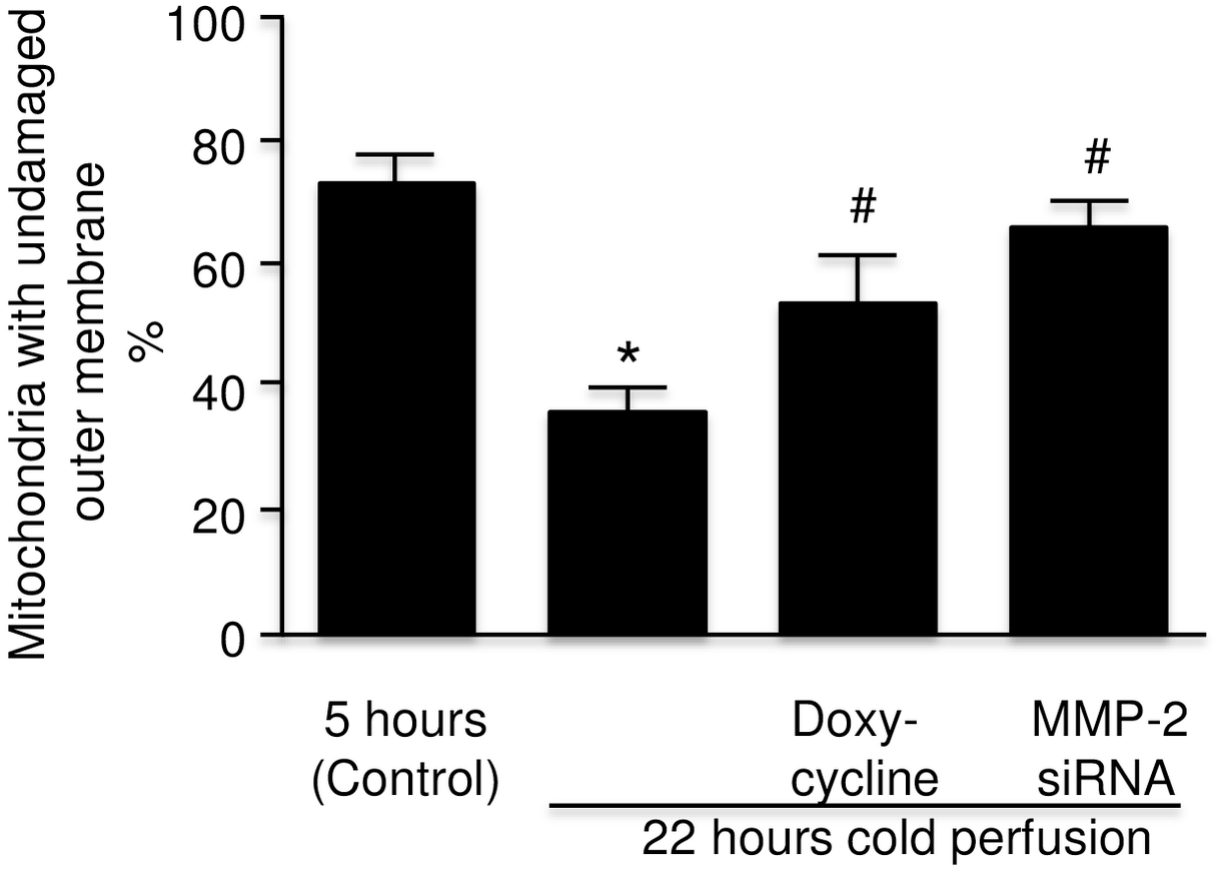
Level of undamaged mitochondria measured by the levels of cytochrome c oxidase activity in rat kidney *tissue* after initial flush and following 22 hours of machine cold perfusion with KPS-1, KPS-1 with doxycycline, and KPS-1 with MMP-2 siRNA (n = 4). Error bars represent Standard Error of the Mean (SEM). * p<0.05 compared to 0 hours using Student’s T test.# p<0.05 compared to 22 hours using Student’s T test.KPS-1 = Kidney Preservation Solution 1MMP = matrix metalloproteinase.

Because of the changes in mitochondria observed on electron microscopy consistent with the role of mitochondria in injury noted by other groups [20], the percentage integrity of the outer membranes of mitochondria was measured in kidneys perfused with and without inhibitors of MMP. A decreased level of undamaged outer mitochondrial membranes was observed in the kidneys after 22 hours of cold perfusion compared to the control level whereas kidneys perfused with DOXY or MMP-2 siRNA showed significant protection of this membrane (Fig 12).

## Discussion

In both human and animal studies, our data show that proteins are released during cold perfusion, including MMP-2 and MMP-9. Markedly higher levels of total protein, NGAL, and LDH were seen in perfusate from cDCDD, compared to DBD human donor kidneys. This is in spite of a much shorter time for cDCDD donor kidney perfusion versus DBD donor perfusion (10.3 vs. 15.9 hours on average, p = 0.02). CcO was not significantly higher in the cDCDD perfusate, but this may have to do with the fact that the perfusion time was much less for cDCDD kidneys than it was for DBD kidneys. Markedly higher MMP-2 and MMP-9 release into the perfusate was also seen in cDCDD donors compared to DBD donors.

In a small animal model of cold perfusion, we found that inhibition of MMP-2 during perfusion using MMP-2 siRNA led to a decrease in MMP-2 level in perfusate but also a decrease in LDH, NGAL, and CcO release after 22 hours of perfusion to a level similar to that seen at 5 hours. The use of the non-specific MMP inhibitor, DOXY, in the perfusion solution led to even greater decreased levels, suggesting that MMPs other than MMP-2 are also involved in the injury.

In the electron micrographs of rat kidneys with warm ischemic injury and machine cold perfusion, changes were seen in mitochondria (swelling and loss of definition of cristae). This was supported by the CcO in the actual rat kidney tissue at 22 hours and the CcO levels in perfusate that increased with time. Indeed, while MMPs are generally thought of as acting on extracellular matrices, they also have been shown to have intracellular actions as well, including on the mitochondria [21].

Many previous studies have documented the role of MMP’s in ischemic damage in the heart [4,8,9,10,11] and it seems reasonable that similar mechanisms are at work in the kidney. Other groups have studied the perfusate from human kidneys for transplantation, with mixed results [22,23]. Our approach was unique, however, in that we utilized an animal model in conjunction with the clinical study of human kidneys. With the animal model, we could modify the cold perfusion solution, control the cold ischemic time, and work with more homogeneous groups of kidneys compared to the clinical setting.

Based on our study, it is not possible to conclude on the mechanism whereby MMPs led to an increase of injury markers, however we might speculate based on the electron micrograph appearance and what is known about MMPs that the effect of MMPs on extracellular matrices and on mitochondria may be responsible.

Our results suggest that certain pharmaceutical agents such as DOXY added to the machine cold perfusion solution may be helpful in reducing the amount of preservation injury to transplant kidneys. It is important to note that this study addressed preservation injury and not ischemia-reperfusion injury, which will be the topic of future studies.

The fact that a clinically available drug (DOXY) exists that is capable of inhibiting MMP-2 makes it appealing to consider a studying the drug clinically in machine cold perfusion; given that deceased donor kidneys often come in pairs, it is possible to randomize one to the control arm and the other to receive DOXY in the perfusion solution. The paired nature of such a study would allow for a relatively small ‘n’.

A reduction in the injury to cold preserved DCDD kidneys should result in the ability to use some of the marginal donor kidneys that would normally be rejected or discarded. If even 10% of the kidneys normally rejected by a program are able to be used, this will translate into thousands of kidneys worldwide per year, thus releasing thousands of patients from dialysis and shortening wait times significantly.

Although not a main focus of our study, it is interesting that in DGF, the levels of MMP-2 and MMP-9 were approximately double compared to the non-DGF kidneys, while the measured injury markers were not significantly different between the two groups. Given that the mechanism whereby DGF occurs remains elusive, the possibility that MMPs are involved in DGF requires further study [24].

In conclusion, this study suggests that MMP-2 and likely other MMPs play a role in transplant kidney preservation injury, and that some of this injury can be prevented by the addition of doxycycline in the preservation solution.

## Materials and Methods

### Human perfusates

Retrieved kidneys were connected to the LifePort^®^ Machine Cold Perfuser (Organ Recovery Systems, Itasca) and perfused with Kidney Perfusion Solution-1 (KPS-1) at 3–5°C using the standard LifePort^®^ settings (pressure 30 mmHg). After the kidney perfusion was terminated, 60–80 mL of the solution was collected and stored at -80°C.

Twenty-four samples (15 DBD and 9 cDCDD) of perfusate from human kidneys were analysed. The standard definition of DGF was used: the need for dialysis in the first week post-transplant. Slow Graft Function (SGF) was defined as serum Cr > 265 μmol/L on post-transplant day 5 and no need for dialysis by day 5 [25].

### Preparation of protein from perfusates for analysis

Proteins were precipitated with Ceramic Hydroxyapatite beads (CHT, Bio-Rad, Hercules, CA, USA) in 2.5 mM phosphate buffer with pH 7.0. The bounded proteins were released from CHT beads with 0.5 M phosphate buffer with pH 7.0.

### Rat kidney model

**Fig 13:**
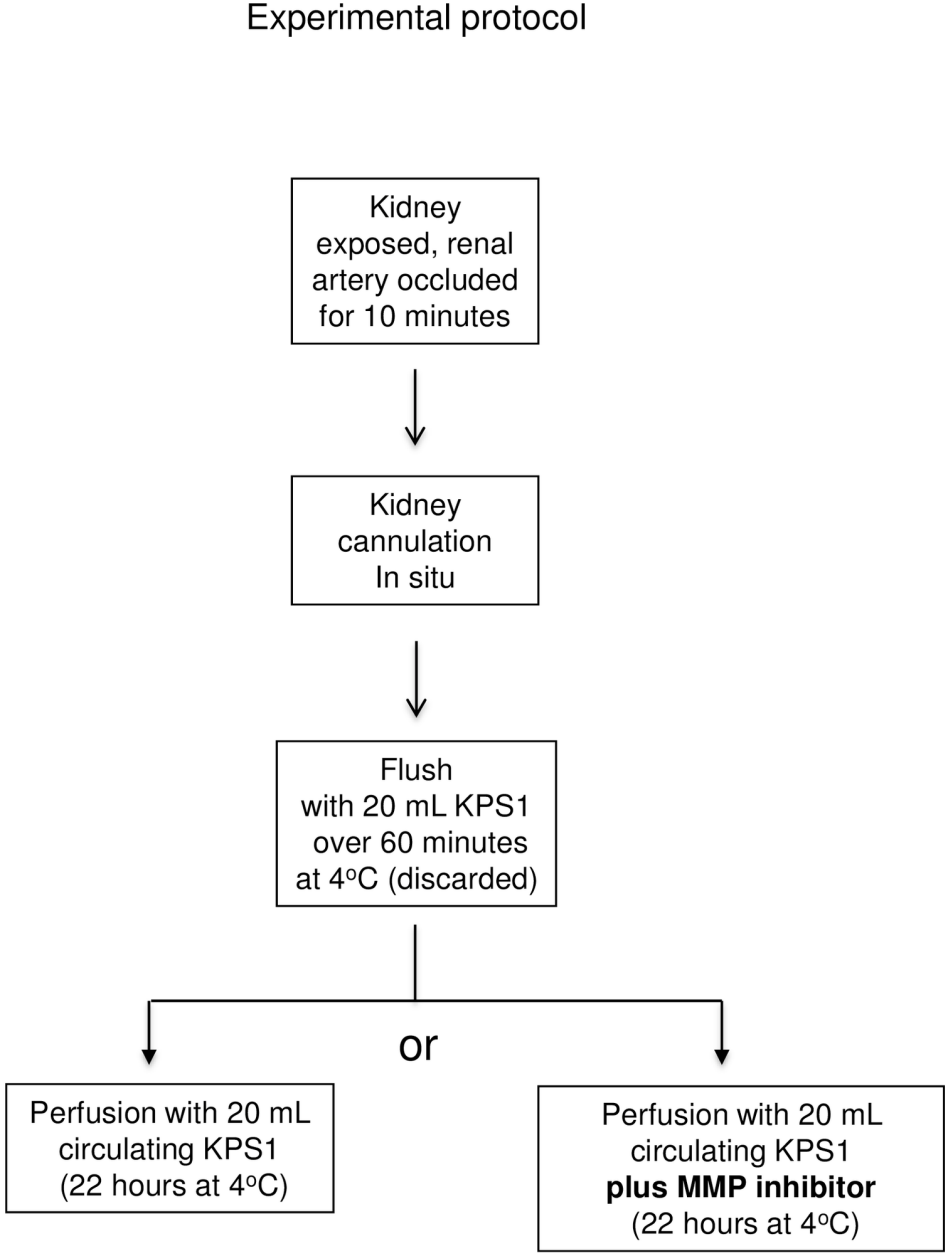
Experimental ProtocolKPS-1 = Kidney Perfusion Solution -1 MMP = matrix metalloproteinase.

The experimental protocol for perfusion of rat kidney is shown in Fig 13. Male Sprague-Dawley rats (200–250 grams, Charles River, Burlington) (4–7 rats per group) were anesthetized with isofluorane. The left renal artery was ligated in situ for 10 minutes of warm ischemia, then cannulated and the kidney removed and rapidly cooled to 4°C. The kidney and tubing were connected to the perfusion apparatus and flushed over 60 min with 20 mL of KPS-1 solution which was then discarded. In keeping with the Canadian Council on Animal Care principle of minimizing the numbers of animals used, other organs were removed in some cases for another experiment, after the kidney was removed. The animal was then sacrificed by exsanguination.

The machine cold perfusion apparatus consisted of a micro-pump (Mini Pulse 3, Gilson) with flow rate of 0.5 mL/min, operating in a 4°C cold room. The KPS-1 solution (20 mL with or without additive) was continuously infused via the renal artery and the effluent passively drained via the renal vein to be recirculated via the pump.

The kidney was perfused for 22 hours; this time was chosen as longer than the usual cDCDD preservation time in order to increase the chances of having significant injury to the rat kidney. Samples of 0.5 mL were collected at 5, 10, and 22 hours and stored at -80°C. At the end of the perfusion, the kidney was stored at -80°C.

### Rat kidney tissue studies

For biochemical studies, frozen kidney tissue was homogenized on ice in 150 mM NaCl, 50 mM Tris-HCl (pH 7.4) containing protease inhibitor cocktail and 0.1% Triton X-100. Homogenates were centrifuged at 10,000 g for 10 min, and the supernatant was stored at -80°C until further use.

### Measurement of NGAL level

NGAL level was measured using ELISA kit (Abcam, Toronto), and expressed as μg of protein per mL of perfusate.

### Measurement of LDH activity

LDH activity was measured by LDH activity assay (Sigma-Aldrich, St. Louis, MO). Enzyme activity is expressed as the amount of NADH generated from the conversion of lactate into pyruvate.

### Measurement of CcO activity

CcO activity was measured using activity assay (Sigma-Aldrich, St. Louis, MO).

### Measurement of the mitochondrial integrity

CcO is located on the inner mitochondrial membrane [26]. The integrity of the outer mitochondrial membrane was calculated, according to the manufacture’s instruction (Sigma-Aldrich, St. Louis, MO), as the ratio of CcO activity in the presence and absence of n-dodecyl β-D-maltoside (detergent).

### Measurement of protein

Protein content of perfusates and the kidney extract in homogenized buffer was measured with the Bradford protein assay (Bio-Rad, Hercules, CA).

### Measurement of MMP levels

Gelatin zymography was performed as previously described [11,27]. After electrophoresis, gels were scanned and MMP levels were measured using Quantity One 4.6 software (Bio-Rad, Hercules, CA). Uncropped gels for Figs 2, 6, 8 and 10 are included in S1 Fig.

### Light microscopy

Sections (4 μm) of formalin-fixed and paraffin-embedded tissues were stained with Periodic Acid Schiff’s stain [28,29] then reviewed in blinded fashion by both a veterinary and a human renal pathologist.

### Electron microscopy

Kidneys (n = 3 each for time zero and time 22 hours) were flushed with glutaraldehyde and 1 mm cubes of tissue were excised from the upper pole in each kidney for electron microscopy, post fixed in osmium tetroxide, dehydrated in graded ethanol, and embedded in an epoxy resin. Ultrathin sections were cut and stained with uranyl acetate and lead citrate and random areas photographed using digital transmission electron microscopy (Hitachi H9500, Tokyo). These were reviewed in blinded fashion by a renal pathologist.

### Inhibition of MMP-2 with DOXY

A concentration of DOXY of 100 μM was used in the zymography gels documenting the inhibitory effect of DOXY and this was the dose used in the therapeutic arm of the rat kidney perfusions. This concentration was used in numerous studies in our lab of MMP inhibition in cardiac ischemia and found to be effective [2].

### Inhibition of MMP-2 with siRNA

A stock solution of a mixture containing a pool of 3 target-specific 19–25 nucleotide small interfering RNAs designed to knock down rat MMP-2 gene expression (Santa Cruz Biotechnology, Santa Cruz, CA, USA) was used (10 μM concentration in a buffer containing 10 μM Tris-HCl, 20 mM NaCl, and 1 mM EDTA at pH 8.0). As a control, scrambled siRNA (Santa Cruz Biotechnology, Santa Cruz, CA) was used.

Perfusion of rat kidney was with 20 ml of KPS-1 containing MMP-2 siRNA or scrambled siRNA at concentration of 50 nM. The kidney was perfused for 22 hours and perfusate samples were collected as described above.

### Statistics

Student’s t test or Chi-squared tests were used as appropriate.

### Study approval

This study conforms to the Guide to the Care and Use of Experimental Animals of the Canadian Council on Animal Care. The protocol was approved by the Animal Care and Use Committee of the University of Saskatchewan (certificate # 20130073). All animal surgery was performed under isofluorane anesthesia, and all efforts were made to minimize suffering.

The protocols involving human perfusates were approved by the University of Saskatchewan Biomedical Research Ethics Board (Bio # 12–44) and the Western University Health Sciences Research Ethics Board (HSREB # 101899). The perfusate that was collected for this study was fluid that is ordinarily discarded and is relevant to neither the donor nor the recipient, so the need for patient consent was waived.

## Supporting Information

S1 Fig Uncropped gels for Figs 2, 6, 8 and 10. (TIF)
