# Supplementary figures and images for: Protection of the Transplant Kidney from Preservation Injury by Inhibition of Matrix Metalloproteinases
Source: PLoS One. 2016 Jun 21;11(6):e0157508. doi: 10.1371/journal.pone.0157508 (PMC4915675; doi:10.1371/journal.pone.0157508)

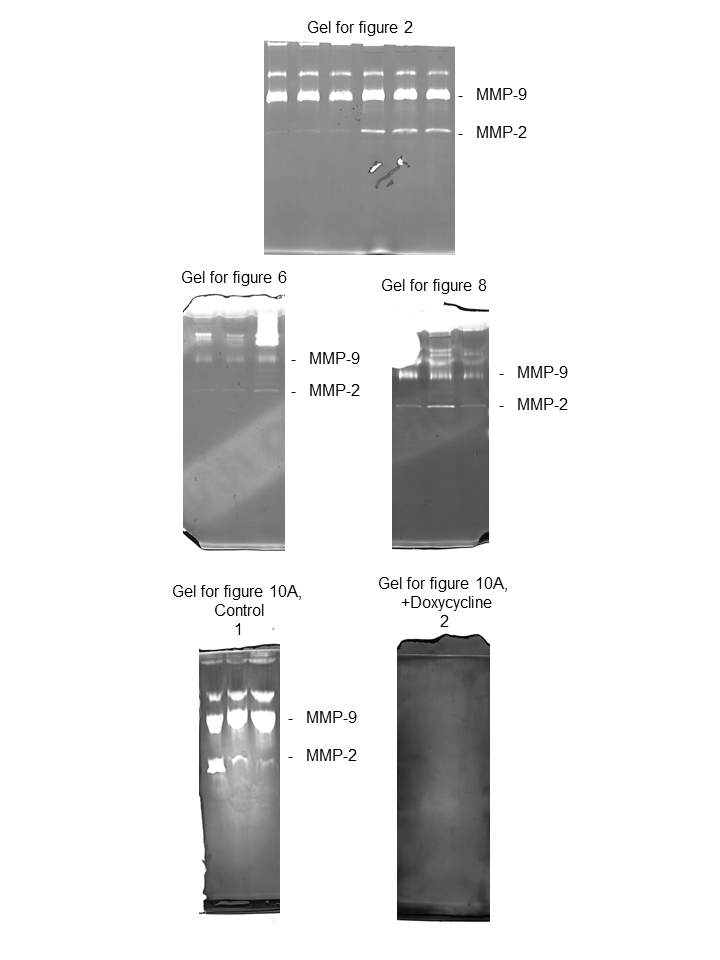

Supplement: S1 Fig — (TIF) [file pone.0157508.s001.tif]
